# Guillain-Barre syndrome associated with hemorrhagic fever with renal syndrome in China: a case report

**DOI:** 10.1186/s12879-018-3049-1

**Published:** 2018-03-27

**Authors:** Jie Jiao, Lei Wu, Jianyuan Yin, Xiaojiao Quan, Wei Chen, Jie Hu

**Affiliations:** 1 Critical Care Medicine, Hainan Branch of Chinese PLA General Hospital, Haitangwan District, Sanyan, 572000 Hainan Province China; 2 Neurology Department, Chinese PLA General Hospital, 28thFuxing Road, Haidian District, Beijing, 100853 China; 3 Emergency Department, Hainan Branch of Chinese PLA General Hospital, Haitangwan District, Sanyan, 572000 Hainan Province China; 4 Critical Care Medicine, Chinese PLA General Hospital, 28thFuxing Road, Haidian District, Beijing, 100853 China

**Keywords:** Hantavirus, Hemorrhagic fever with renal syndrome, Acute kidney injury, Guillain-Barre syndrome

## Abstract

**Background:**

We describe a case of Guillain-Barre syndrome (GBS) associated with hemorrhagic fever with renal syndrome. To our knowledge, only five cases of GBS associated with Hantavirus infection have been reported so far.

**Case presentations:**

A 62-year-old man presented intermittent fever, chill and oliguria. According to remarkable leukocytosis, atypical lymphocytes, thrombocytopenia and former dwelling in hemorrhagic fever-endemic area, he was suspected as hemorrhagic fever with renal syndromeand certified with positive Hantavirus IgG. Later, the patient had symmetrical flaccid paralysis of all extremities. Electromyography showed peripheral nerve injury (mainly in axon). The patient was diagnosed as having acute motor sensory axonal neuropathy (AMSAN). After immunoglobulin infusion, patient showed progressive recovery and was transferred 3 weeks after his first admission to a rehabilitation center.

**Conclusions:**

Our case was the 6th reported case of GBS associated with hemorrhagic fever with renal syndrome. Moreover, we for the first time classified the subtype of GBS (AMSAN) based on the electrophysiology characteristics. GBS should be suspected in patients who are already diagnosed as hemorrhagic fever with renal syndrome when delayed symmetrical limb paralysis occurs. Until recent now, GBS was only reported in hemorrhagic fever patients in Europe and Asia, which termed as hemorrhagic fever with renal syndrome.

## Background

Hantaviruses are rodent-borne zoonotic viruses that cause two life-threatening clinical syndromes in human beings: hemorrhagic fever with renal syndrome (HFRS) in Asia and Europe, and Hantavirus cardiopulmonary syndrome in America [1]. Classic HFRS occurs in five phases: fever, hypotension, oliguria, polyuria, and convalescence. Sequelae are not common but include chronic renal disease and hypertension. Extrarenal manifestations include acute impairment of visual function, acute myopia, central nervous system complications with seizures, myocarditis, and severe gastrointestinal hemorrhages [2].

Guillain-Barre syndrome (GBS) is an acute inflammatory immune-mediated polyradiculoneuropathy, sometimes with no sepcific causes [3]. Rencently, clinical manifestations and electrophysiology characteristics play vital roles in GBS diagnosis, clinical subtypes classification and prognosis prediction [4]. The underlying electrophysiology abnormalities of GBS and its subtypes were categorized as demyelination, axonal or unclassifiable based on the criteria [5, 6]. To our best knowledge, only five cases of GBS associated with Hantavirus infection have been reported so far [7–11]. Here, we describe the case of a 62-year-old man who developed GBS while recovering from Hantavirus infection.

## Case presentation

A 62-year-old man came to our emergency for intermittent fever, chill, oliguria over the past 4 days. The patient lived in a rural area of Heilongjiang Province and newly came to Hainan Province 2 weeks ago. His past medical history was marked by a subtotal gastrectomy 4 years ago. There was no history of cardiac or pulmonary disease.

Physical examination (day4) showed tachycardia, tachypnea, subconjunctival hemorrhage in the right eye and scattered ecchymosis on the chest. Blood tests showed WBC 71.12 × 10^9^/L (3.5–10 × 10^9^/L),Hb 194 g/L (139–179 g/L),PLT 15 × 10^9^/L (100–300 × 10^9^/L), ALT 220.6 U/L (0–40 U/L), AST 446.7 U/L (0–40 U/L), Cr 365 μmol/L (30-110 μmol/L), BUN 19.8 mmol/L (1.8–7.5 mmol/L), BNP 1422 pg/mL (0–150 pg/mL), PCT 13.26 ng/mL (< 0.05 ng/ml). Blood gas demonstrated severe metabolic acidosis. Cardiac ultrasound was normal except slight left ventricular hypertrophy. Abdominal and pelvic CT scan showed ascites and pelvic effusion. Pulmonary CT scan showed pulmonary atelectasis of right lower lobe and bilateral pleural effusion. He was suspected as hemorrhagic fever with renal syndromeaccording to remarkable leukocytosis, atypical lymphocytes, thrombocytopenia, acute kidney injury and former dwelling in hemorrhagic fever-endemic area and unknown infectious site and was later certified with positive Hantavirus IgG. Dengue fever was ruled out by Dengue fever IgG and IgM dectection, while Malaria was eliminated by blood smear. Two days later (day 6), the patient deteriorated and presented with respiratory failure (respiratory distress and mechanical ventilation), refractory shock and severe metabolic acidosis. He was admitted to ICU and infused with sedative, analgesic and muscle relaxant.

On day 12, after cease of sedation, analgesia and muscle relaxants, the patient had symmetrical flaccid paralysis of all extremities but normal spontaneous respiration. Neurological physiological examination revealed consciousness, quadriplegic with sensorial disorder of extremity terminal nerve, decreased muscle tension, tendon areflexia, without nystagmus, diplopia or facioplegia and Babinski sign. Thus, the patient was suspected as GBS. We utilized electromyography (EMG) to confirm the diagnosis. Data showed(1) abnormal bilateral peroneal nerve motor conductions, decreased compound muscle action potential (CMAP) amplitude of right median nerve and ulnar nerve but normal motor nerve conduction velocity (MNCV); (2) decreased sensory nerve action potential (SNAP) amplitude of right median nerve and peroneal nerve but normal sensory nerve conduction velocity (SNCV); (3) F wave absence of right tibial nerve and prolonged distal moter latency (DML) of left tibial nerve; (4) several spontaneous electromyographic activity in right tibialis anterior muscle, quadriceps femoris and first dorsal interosseus, indicating acute motor sensory axonal neuropathy (AMSAN), a peculiar type of GBS.

**Fig. 1:**
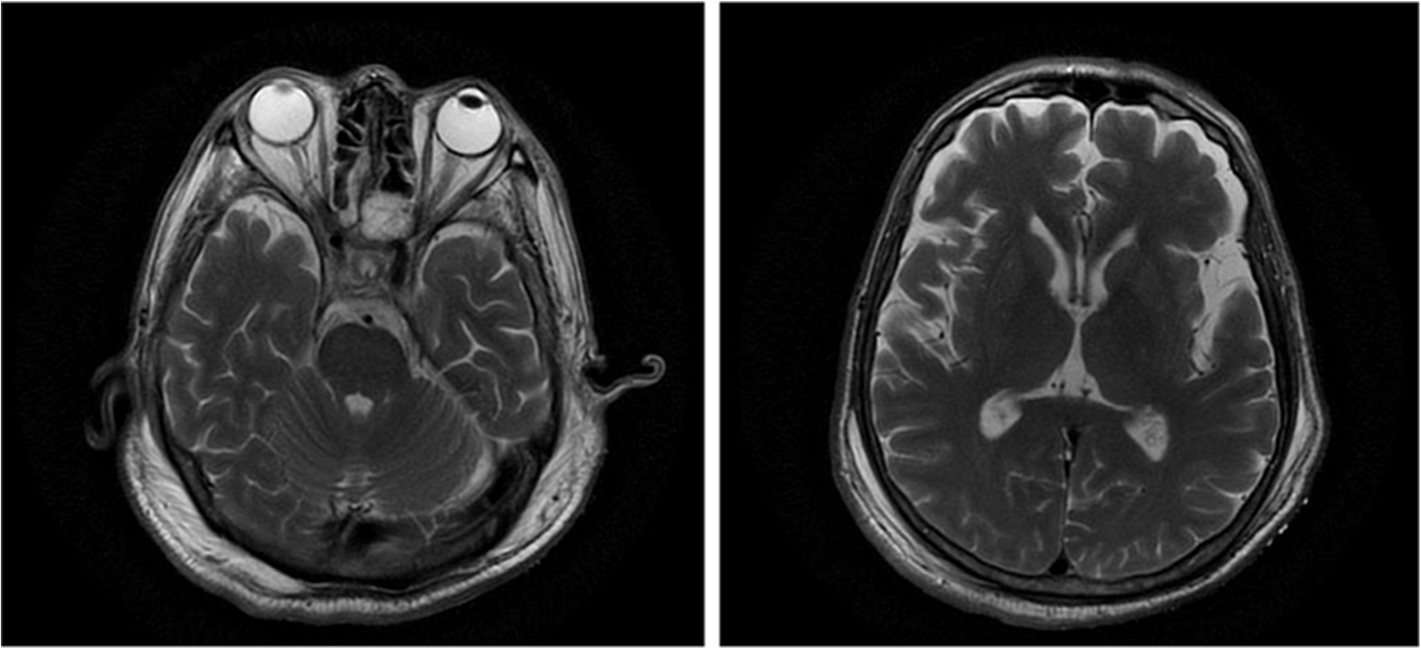
Brain MRI showing normal signal in mesocephalon (left) and basal ganglia region (right) in T2-weighted

Since we noted a marked increase of the serum sodium level (precipitously increased from 144 mmol/L to 157 mmol/L on day 10 when urine volume remarkably raised to more than 9000 ml), an MRI was performed to eliminate the potential differential diagnosis of osmotic demyelination syndrome (ODS) after paralysis onset. Brain MRI showed no abnormal signal in mesocephalon or basal ganglia region on T2-weighted (Fig. 1).

Treatment was initiated with intravenous immunoglobin (0.4 g/kg per day) for 5 days without complications. Afterwards, the patient showed progressive recovery with improved myodynamia and was transferred 3 weeks after his first admission to a rehabilitation center. He was able to walk with minimal help 6 months later.

## Discussion

GBS is the most common and severe acute paralytic neuropathy [12]. The acute progression of limb weakness, often accompanied with sensory and cranial nerve involvement 1–2 weeks after immunological stimulation, proceeds to its peak clinical deficit in 2–4 weeks [13] is important. The disorder of examination of the cerebrospinal fluid (CSF) is classically known for its cytoalbuminological dissociation—the combination of a normal cell count and increased protein level. Nerve conduction studies (NCS) can help the doctors to make the diagnosis and enable them to divide GBS into acute inflammatory demyelinating polyneuropathy, acute motor axonal neuropathy, or acute motor and sensory axonal neuropathy [14]. Early initiation of intravenous immunoglobulins (IVIg) [15] or plasma exchange (PE) [16] proved to be benefit and crucial, especially in patients with rapidly progressive weakness. However, there is no evidence for superiority in the efficacy or safety of IVIG or PE in the management [17].

In our case, GBS was suspected once the patient was found as having symmetrical flaccid paralysis of all limbs 12 days after fever onset. Although we did not get the proof from CSF devoid of consent from patient or his relatives, electromyography demonstrated peripheral nerve injury, major in axon, indicating AMSAN, a peculiar type of GBS. Thus, IVIG therapy was immediately initiated, contributing almost full recovery of neurological function.

As far as I know, only five similar cases have been reported so far. In 1992, Forslund et al. reported the first case presenting polyradiculitis similar as GBS accompanied with nephropathia epidemica [10] in Finland. In 1993, a second case of Hantavirus infection and collateral GBS was reported by Esselink [7]. A woman manifested fever, oliguria and numbness in her hands and feet on admission. After only 2 days, she developed severe paresthesia and weakness of extremities, ataxia and areflexia. Hantavirus specific antibodies were detectable both in the serum and the CSF. The EMG characteristics were in keeping with GBS. The clinicians concluded that the patient had GBS associated with Hantavirus infection based on the above clinical manifestations. In 1995, a third case was reported in France. A woman presented fever, myalgia and showed cytoalbuminological dissociation in CSF examination on admission. After 5 days, the patient developed ascending paresthesia and paresis with areflexia. EMG study met the criteria of GBS. However, Hantavirus specific antibodies were not detectable on the first serum but turned positive later, which further confirmed the diagnosis [8]. The latest case was reported in 2014 in Belgium. A 62-year-old man was admitted for fever, headache and diffuse myalgia. The HFRS was diagnosed immediately and the patient’s condition deteriorated with severe myasthenia, progressive weakness of extremities, areflexia and distal paresthesia. CSF examination showed an increased concentration of protein without white cells. EMG study was suggestive of GBS but Hantavirus serologic test on the CSF was negative. After IVIG therapy, the patient fully recovered [9].

It was worth to mention another case from Korea in 2016. Lim et al. reported a 44-year-old male logger complained of acute quadriplegia and dyspnea. Mechanical ventilation was immediately initiated. He was an HBV carrier with mild liver injury as referred as hepatic enzyme elevation, and positive for Hantavirus antibody. He was confirmed as GBS and IVIG therapy was administered. Eight months later, quadriplegia and hypesthesia recurred. However, IVIG therapy at this time did n’t work, while steroid therapy had some effect. A diagnosis of chronic inflammatory demyelinating polyneuropathy (CIDP) was made. Two months later, severe extremity pain and dyspnea developed again, thus steroid pulse therapy was initiated. In conclusion, patients both infected with HBV and Hantavirus in whom GBS has been initially diagnosed should be followed up for a long time, on account of the possibility of recurrence or deterioration, and acute-onset CIDP should always be taken into consideration [11].

Interestingly, all of the six patients came from Europe or Asia, indicating that GBS might be only associated with HFRS rather than HPS after Hantavirus infection. It was inferred that different pathogenic Hantavirus Serotype might favor different cell types (neurons or alveolar epithelium).

Given the history of precipitously increment of serum sodium, osmotic demyelination syndrome (ODS) was also suspected after paralysis onset. ODS was demonstrated as an uncommon neurological disorder caused by damage to the myelin sheath of brain cells [18]. It has traditionally been described in alcoholics or in patients with rapid osmolar shifts (particularly with a precipitous rise in serum sodium concentration) [19]. Magnetic resonance imaging (MRI) has led to a greater recognition of ODS even with mild and asymptomatic cases. The typical radiological manifestations are hyperintense lesions in the central pons or extrapontine structures on T2-weighted and fluid-attenuated inversion recovery sequences with correlated hypointensity on T1-weighted sequences. In our case, brain MRI showed normal signal in mesocephalon and basal ganglia region in T2-weightedand thus exclude the diagnosis.

There were some limitations in our case. CSF examination was absent since we did not get consent from the patient’s daughter. However, according to the recently proposed new diagnostic classification system, GBS diagnosis could be decided with clinical ground and electrophysiological examinations, without the need for additional laboratory tests, and whether or not they fulfill existing diagnostic criteria [20]. Moreover, the sodium level changed precipitously during the polyuria phase, which perplexed the diagnosis of GBS with ODS. In that case, brain MRI was performed and later excluded the possibility of ODS.

## Conclusion

In summary, we describe a case of GBS (AMSAN) associated with hemorrhagic fever with renal syndrome. To our knowledge, only five cases of GBS associated with Hantavirus infection have been reported. Moreover, we for the first time classified the subtype of GBS (AMSAN) based on the electrophysiology characteristics. Until recent now, GBS was only reported in hemorrhagic fever patients in Europe and Asia, which termed as hemorrhagic fever with renal syndrome. It promoted physicians in Europe and Asia to pay attention to neurological function of patients who are already diagnosed as hemorrhagic fever with renal syndrome especially when delayed symmetrical paralysis occurs. Early management may avoid permanent neurological injury.

## Abbreviations

ALT: Alanine aminotransferase
AMSAN: Acute motor sensory axonal neuropathy
AST: Aspartate aminotransferase
BNP: Brain natriuretic peptide
BUN: Blood urea nitrogen
CIDP: Chronic inflammatory demyelinating polyneuropathy
CMAP: Compound muscle action potential
Cr: Creatinine
CSF: Cerebrospinal fluid
CSF: Cerebrospinal fluid
CT: Computed tomography
DML: Distal moter latency
EMG: Electromyography
GBS: Guillain-Barre syndrome
Hb: Hemoglobin
HBV: Hepatitis B virus
HFRS: Hemorrhagic fever with renal syndrome
HPS: Hantavirus pulmonary syndrome
IgG: Immunoglobulin G
IVIG: Immunoglobulins
MNCV: Motor nerve conduction velocity
NCS: Nerve conduction studies
ODS: Osmotic demyelination syndrome
PCT: Procalcitonin
PE: Plasma exchange
PLT: Platelets
SNAP: Sensory nerve action potential
SNCV: Sensory nerve conduction velocity
WBC: White blood cells

## References

[1] Mattar S, Guzman C, Figueiredo LT (2015). Diagnosis of hantavirus infection in humans. Expert Rev Anti-Infect Ther.

[2] Muranyi W, Bahr U, Zeier M, van der Woude FJ (2005). Hantavirus infection. J Am Soc Nephrol.

[3] Esposito S, Longo MR (2017). Guillain-Barre syndrome. Autoimmun Rev.

[4] Hiew FL, Ramlan R, Viswanathan S, Puvanarajah S (2017). Guillain-Barre syndrome, variants & forms fruste: reclassification with new criteria. Clin Neurol Neurosurg.

[5] Rajabally YA, Durand MC, Mitchell J, Orlikowski D, Nicolas G (2015). Electrophysiological diagnosis of Guillain-Barre syndrome subtype: could a single study suffice?. J Neurol Neurosurg Psychiatry.

[6] Hadden RD, Cornblath DR, Hughes RA, Zielasek J, Hartung HP, Toyka KV, Swan AV (1998). Electrophysiological classification of Guillain-Barre syndrome: clinical associations and outcome. Plasma exchange/Sandoglobulin Guillain-Barre syndrome trial group. Ann Neurol.

[7] Esselink RA, Gerding MN, Brouwers PJ, Solleveld H, Jordans JG, Groen J, Osterhaus AD (1994). Guillain-Barre syndrome associated with hantavirus infection. Lancet (London, England).

[8] Rabaud C, May T, Hoen B, Maignan M, Gerard A, Canton P (1995). Guillain-Barre syndrome associated with hantavirus infection. Clin Infect Dis.

[9] Tassart G, Balbeur S, Deltombe T, Tintillier M, Cuvelier C (2014). Guillain-Barre syndrome associated with Puumula hantavirus infection. Acta Clin Belg.

[10] Forslund T, Saltevo J, Anttinen J, Auvinen S, Brummer-Korvenkontio M, Korhonen A, Poutiainen M (1992). Complications of nephropathia epidemica: three cases. J Intern Med.

[11] Lim JY, Lim YH, Choi EH (2016). Acute-onset chronic inflammatory demyelinating polyneuropathy in hantavirus and hepatitis B virus coinfection: a case report. Medicine.

[12] Willison HJ, Jacobs BC, van Doorn PA (2016). Guillain-Barre syndrome. Lancet (London England).

[13] Fokke C, van den Berg B, Drenthen J, Walgaard C, van Doorn PA, Jacobs BC (2014). Diagnosis of Guillain-Barre syndrome and validation of Brighton criteria. Brain.

[14] Ho TW, Mishu B, Li CY, Gao CY, Cornblath DR, Griffin JW, Asbury AK, Blaser MJ, McKhann GM (1995). Guillain-Barre syndrome in northern China. Relationship to campylobacter jejuni infection and anti-glycolipid antibodies. Brain.

[15] Hughes RA, Swan AV, van Doorn PA: Intravenous immunoglobulin for Guillain-Barre syndrome. Cochrane Database Syst Rev 2014(9):CD002063. 10.1002/14651858.CD002063.plus6. 10.1002/14651858.CD002063.pub6 PMC6781841 25238327

[16] Chevret S, Hughes RA, Annane D (2017). Plasma exchange for Guillain-Barre syndrome. Cochrane Database Syst Rev.

[17] Ortiz-Salas P, Velez-Van-Meerbeke A, Galvis-Gomez CA, Rodriguez QJ (2016). Human immunoglobulin versus plasmapheresis in Guillain-Barre syndrome and myasthenia gravis: a meta-analysis. J Clin Neuromuscul Dis.

[18] Brown WD (2000). Osmotic demyelination disorders: central pontine and extrapontine myelinolysis. Curr Opin Neurol.

[19] Singh TD, Fugate JE, Rabinstein AA (2014). Central pontine and extrapontine myelinolysis: a systematic review. Eur J Neurol.

[20] Wakerley BR, Uncini A, Yuki N, Group GBSC, Group GBSC (2014). Guillain-Barre and miller fisher syndromes--new diagnostic classification. Nat Rev Neurol.

